# Amoxicillin-induced bacterial gut dysbiosis decreases *IL-1β* and *IL-6* expression but exacerbate lung inflammatory response against *Mycobacterium bovis*—Bacille Calmette-Guérin (BCG)

**DOI:** 10.1371/journal.pone.0319382

**Published:** 2025-02-26

**Authors:** Tatimara M. Miyauchi-Tavares, Evandro Neves Silva, Joyce Alves dos Santos, Priscila V. Sousa, Marcos F. Teodoro Braga, Caroline M. Carminatti, Victoria B. Lanza, Bruna C. Fagundes, Rômulo Dias Novaes, Leonardo Augusto de Almeida, Patrícia Paiva Corsetti

**Affiliations:** 1 Laboratory of Molecular Biology of Microorganisms, Federal University of Alfenas (UNIFAL), Brazil; 2 Department of Structural Biology, Federal University of Alfenas (UNIFAL), Alfenas, Brazil; Cornell University, UNITED STATES OF AMERICA

## Abstract

Tuberculosis is one of the leading causes of global mortality, and the standard, prolonged, and intensive treatment can affect intestinal homeostasis. This study investigated amoxicillin-induced bacterial gut dysbiosis and its impact on the immune response of C57BL/6 mice to pulmonary infection by *Mycobacterium bovis*—BCG. It was observed that amoxicillin treatment resulted in bacterial gut dysbiosis, characterized by an increase in the phylum Proteobacteria and a reduction in Bacteroidetes and Firmicutes. This alteration was associated with a decrease in the animals’ body weight and a reduction in the expression of pro-inflammatory cytokines such as *IL-1β* and *IL-6*, suggesting a compromised immune response. Additionally, microstructural analysis revealed significant alterations in the caecum and pulmonary structure of the mice, indicating tissue damage associated with intestinal dysbiosis. The results indicate that amoxicillin-induced bacterial gut dysbiosis may negatively affect pulmonary immunity and exacerbate *M. bovis*-BCG infection, highlighting the need to consider the impacts of intestinal microbiota on the development and control of tuberculosis. This study contributes to the understanding of the interaction between intestinal microbiota, antibiotic treatment, and immunity in pulmonary infections.

## 1. Introduction

Tuberculosis (TB), caused by bacteria of the genus *Mycobacterium*, continues to be one of the greatest public health challenges globally due to its high morbidity and mortality [1], being the leading cause of death from a single pathogen infection [2]. According to the World Health Organization (WHO), in 2022, approximately 7.5 million people were diagnosed with TB, and 1.3 million people died from the disease. Clinically, TB manifests with a productive cough, which may or may not contain blood, afternoon fever, night sweats, asthenia, and weight loss [3]. While *Mycobacterium tuberculosis* is the etiological agent of TB in humans, *Mycobacterium bovis* is responsible for cattle and other mammalian infection and can also infect humans. Transmission occurs through the inhalation of contaminated aerosols or the consumption of unpasteurized dairy products [4]. The standard treatment for *Mycobacterium* spp. is complex and prolonged, involving the use of four drugs for a minimum of six months for humans: isoniazid and rifampicin throughout the period, with ethambutol and pyrazinamide added in the first two months [3]. Due to the excessive and prolonged use of these medications, intestinal homeostasis may be at risk. The gut microbiota is a community of microorganisms that symbiotically inhabit the intestinal lumen [5], and one of its functions is to regulate both adaptive and innate immunity by producing small molecules (metabolites) that influence the threshold of immune activation. Specific species of the gut microbiota have been shown to induce different immunological phenotypes or cytokine responses that can influence the pathogenesis or pathology of the disease, associating with the elimination or control of *Mycobacterium* spp. in the context of tuberculosis, which requires a coordinated and balanced expression of subsets of T cells [2].

In this spectrum, dysregulations in the microbiome that alter this necessary balance are correlated with the early pulmonary colonization of *Mycobacterium* and reduced Th1 immunity in mice, according to Eribo and collaborators [2], indicating a regulatory role of the gut microbiota in pulmonary immunity [6]. The bacterial communities present in the gut microbiota show a high degree of plasticity, with their composition influenced by various environmental and host factors such as diet, age, physiological state, and genetic history [5,7], as well as pathological factors like inflammatory diseases and antibiotic use, which can impact intestinal diversity and composition [8].

Specifically regarding tuberculosis, recent evidence suggests a relationship between dysbiosis and disease development, with the composition of the gut microbiome being altered during the disease and anti-TB drug treatment [2].

In this context, amoxicillin, a β-lactam antibiotic derived from penicillin [9], is widely prescribed for its moderate spectrum and efficacy. However, this medication significantly alters the diversity of the gut microbiota [8,10]. The alterations caused by the use of antibiotics like amoxicillin can be critical for maintaining the balance of the gut microbiota, interfering with its functions and potentially affecting the response to pulmonary colonization by *Mycobacterium* spp. [2]. Given the need for further studies to clarify the relationship between gut microbiota and tuberculosis, the main goal of this study was to evaluate amoxicillin-induced gut dysbiosis and its impact on the murine immune response to pulmonary infection by *Mycobacterium bovis* – Bacille Calmette-Guérin (BCG).

## 2. Materials and methods

### 2.1. Mice model and ethics statement

Male and female C57BL/6 mice, 6 to 8 weeks-old, were housed in individual cages under a 12-hour light/dark cycle, with standard rodent diet ad libitum. This study strictly followed Brazilian mice Experimentation Laws (nº. 6638 and nº. 9605) and was approved by the mice Experimentation Ethics Committee of the José do Rosário Vellano University (CEUA nº. 45A/2017) following in compliance with the ARRIVE (Animal Research: Reporting of In Vivo Experiments) guidelines, as described at https://arriveguidelines.org. Humane endpoints could be applied to minimize unnecessary suffering in mice if necessary. None of the mice exhibited any behavioral changes that would indicate the need to assign them to a humane endpoint or failed to survive the proposed experiment.

### 2.2. Experimental groups and amoxicillin treatment

The mice were randomly assigned to four treatment groups (n = 5): G1 - received a sterile phosphate-buffered saline (PBS) solution by oral gavage for 14 days; G2 - received 500 mg/kg of amoxicillin trihydrate (AMOX) by oral gavage for 14 days; G3 - were infected only with *Mycobacterium bovis—*Bacille Calmette-Guérin (BCG); and G4 - received 500 mg/kg of amoxicillin trihydrate by oral gavage for 14 days and were infected with *M. bovis—*BCG (AMOX +  BCG) on day 15. The mice were weighed daily, their survival was monitored, and their feces were collected and aseptically stored in 1.5-mL sterile tubes on day 15.

### 2.3. *M. bovis*-BCG strain and intratracheal infection of the mice

Strains of *M. bovis* - BCG Moreau, produced by the Ataulpho de Paiva Foundation in Rio de Janeiro, Brazil, were cultivated and maintained in the collection of the Laboratory of Molecular Biology of Microorganisms (LaBioMol) at the Federal University of Alfenas (UNIFAL). The mice were infected intratracheally on the fifteenth day with the *M. bovis* - BCG Moreau strain. Briefly, the mice were anesthetized with ketamine/xylazine (80/10 mg/kg, i.p.), and their tracheas were exposed through a small incision in the skin of the neck. The infection was performed with 1×10^5^ colony-forming units (CFU) of *M. bovis* - BCG. After the infection, the neck incision was sutured. The mice were then kept in a vertical position for 5 minutes and subsequently transferred to a warming pad until full recovery from anesthesia [11]. Twenty-four hours later, all mice from both treatments, infected or not, were euthanized with a lethal dose of ketamine/xylazine anesthetic (300/30 mg/kg, i.p.).

### 2.4. Extraction of fecal DNA and identification of bacterial microbiota by metabarcoding

Total DNA was extracted from fecal samples of the PBS or AMOX group using the QIAamp DNA Stool Mini Kit (QIAGEN Inc). The DNA was quantified using the Invitrogen Qubit Fluorometer (Thermo Fisher Scientific). One hundred nanograms of total DNA were used to evaluate 16S ribosomal RNA (rRNA) sequences on the Illumina HiSeq platform (Illumina). Briefly, 16S rRNA/18S rRNA/ITS genes from distinct regions (16SV4/16SV3/16SV3-V4/16SV4-V5, 18SV4/18S V9, ITS1/ITS2, ArcV4) were amplified using specific primers (e.g., 16SV4: 515F-806R, 18SV4: 528F-706R, 18SV9: 1380F-1510R).

All PCRs were performed with Phusion® High-Fidelity PCR Master Mix (New England Biolabs). Samples between 400 and 450 bp were selected for further experiments. PCR products were mixed in equidensity ratios and purified using the Qiagen Gel Extraction Kit (QIAGEN Inc). Sequencing libraries were generated using the NEBNext® Ultra™ DNA Library Prep Kit for Illumina, following the manufacturer’s recommendations, with index codes added. The library quality was assessed on the Agilent Bioanalyzer 2100 system (Agilent Technologies). Amplicons were sequenced on the Illumina paired-end platform to generate 250-bp paired-end raw reads, which were assembled and pre-treated to obtain Clean Tags. Chimeric sequences in Clean Tags were detected and removed to obtain the final Effective Tags. Negative controls and technical replicates were performed to detect any potential contamination during sample handling and library preparation and to ensured that observed differences in microbial composition were not due to technical artifacts.

Sequence analysis was performed using Uparse software (Uparse v7.0.1001). Sequences with ≥  97% similarity were assigned to the same OTUs. A representative sequence for each OTU was selected for further annotation. The GreenGene Database was used for taxonomic annotation based on the RDP classifier algorithm (version 2.2). To study the phylogenetic relationships of different OTUs and the differences in dominant species between samples (groups), multiple sequence alignment was conducted using MUSCLE software (version 3.8.31) [10,12,13]. OTU abundance information was normalized using a standard sequence number corresponding to the sample with the fewest sequences, and KRONA was used to display the species annotation results.

### 2.5. Real-time RT-PCR for cytokine expression and *M. bovis*-BCG quantification

The right lungs of the mice from both groups were macerated and homogenized in TRIzol reagent (Invitrogen, Life Technologies) to isolate total RNA. Reverse transcription of 1 μg of total RNA was performed using illustraTM Ready-To-Go RT-PCR Beads (GE Healthcare). Real-time PCR (RT-PCR) was conducted in a final volume of 10 μL containing: SYBR® Green PCR Master Mix (Applied Biosystems), oligo-dT or random-primer cDNA as the PCR template, and 20 μmol/L of primers. PCR was carried out on the ABI 7500 Real-Time PCR System (Applied Biosystems) with the following cycling parameters: 60°C for 10 minutes, 95°C for 10 minutes, 40 cycles of 95°C for 15 seconds and 60°C for 1 minute, followed by a dissociation stage of 95°C for 15 seconds, 60°C for 1 minute, 95°C for 15 seconds, and 60°C for 15 seconds. Primers for *IL-1β*, *IL-6*, *IL-10*, and *BCG* were used to amplify specific fragments of 100–120 bp corresponding to specific genetic targets (S1 Table). All data were presented as relative expression units for *IL-1β*, *IL-6*, and *IL-10*, normalized to the *β-actin* gene. For the quantification of *BCG* expression, analysis was performed using the relative quantification of the AMOX +  BCG group compared to the BCG group, using 2-Δct. All PCR measurements were performed in triplicate.

### 2.6. Microstructural analysis

Fragments of the caecum and the left lung were fixed in 10% formaldehyde solution, dehydrated in ethanol, and embedded in paraffin. Histological sections with a thickness of 5 μm were cut and stained with hematoxylin and eosin. Both organs were observed and digital images were captured using a bright-field photomicroscope coupled with ×  20 (magnification ×  200) and ×  100 (magnification ×  1000) objective lenses (Axio Scope A1, Carl Zeiss). For each organ and mice, 5 randomly sampled histological fields were analyzed. The caecum was analyzed at 400 × magnification, and the following parameters were measured: mucosa thickness (µm), mucosa cells (n/mm²), goblet cells (n/mm²), epithelium thickness (µm), crypt length (µm), and intraepithelial lymphocytes (n) [10]. Using the same microscopic magnification, lung samples were analyzed and the following microstructural parameters were quantified: cellularity (n/mm²), alveoli (n/mm²), alveolar surface density (mm²/mm³), septum/alveoli (%), and volume of hemorrhagic foci [13]. All these microstructural parameters were quantified using computational planimetry with the previously calibrated image analysis software Image Pro-Plus 4.5 (Media Cybernetics Inc) [14].

### 2.7. Statistical analysis

The experiments were repeated at least twice with similar results. The figures presented in this work indicate the most representative result obtained. Graphs and statistical analyzes were performed using the GraphPad Prism 8.1 program (GraphPad Software, Inc, La Jolla, CA, USA). The results obtained were expressed as mean and standard deviation (mean ±  SD). Normality in data distribution was assessed using the Kolmogorov-Smirnov test. The variance of the data obtained from both groups investigated was analyzed using one-way analysis of variance (One-way ANOVA) followed by the Student-Newman-Keuls test for multiple comparisons. Non-parametric data were compared using the Kruskal-Wallis test. The confidence level of all tests was set at 95% (p ≤  0.05).

## 3. Results

### 3.1. Amoxicillin-induced bacterial gut dysbiosis increases the abundance of *Proteobacteria* and decreases Bacteroidetes and Firmicutes, leading to reduced body weight of C57BL/6 mice

**Fig 1:**
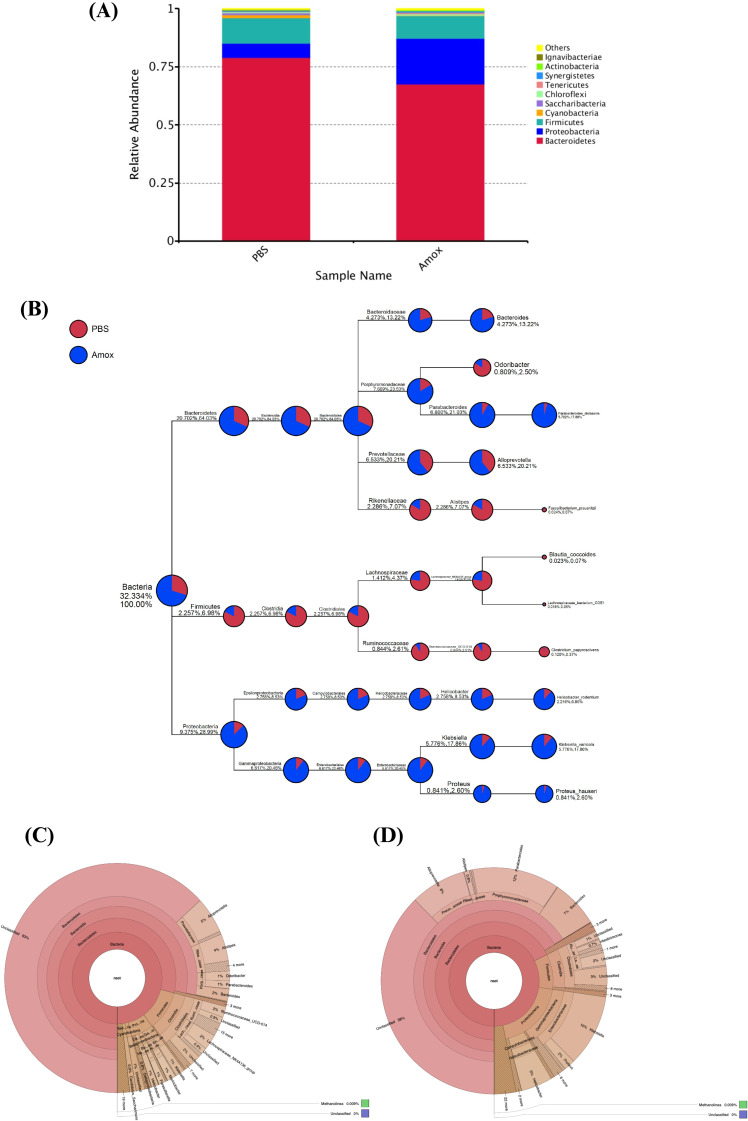
Amoxicillin-induced bacterial gut dysbiosis in mice. (A) Operational Taxonomic Units (OTUs) based on phyla found in the feces of PBS-treated mice and those with AMOX-induced gut dysbiosis. (B) Taxonomic tree of specific species in mice treated with PBS (red) or AMOX (blue). The circle sizes represent the relative abundance of the species. The first number below the taxonomic name indicates the percentage across the entire taxon, while the second number represents the percentage within the selected taxon. **(C)** and (D) Krona analysis of OTUs showing the decrease in Bacteroidetes and Firmicutes populations and the increase in Proteobacteria in the feces of C57BL/6 mice treated with amoxicillin. The Krona classification follows this order: Kingdom, phylum, class, order, family, genus, and species.

To evaluate whether amoxicillin could alter intestinal bacterial diversity, mice were treated for 15 days with AMOX, administered via oral gavage. The bacterial microbiota of these mice’ feces was compared to that of control mice. The metabarcoding sequence analysis revealed an increase in the relative abundance of the phylum Proteobacteria and a decrease in the phylum Bacteroidetes and Firmicutes compared to the mice treated with PBS (Fig 1A). Regarding specific taxa, it was observed that *Proteus*, *Klebsiella*, *Parabacteroides*, and *Helicobacter* genus thrived in the microbiota of dysbiotic mice (Fig 1B).

The high-throughput metagenomic analysis, performed using the Krona software, revealed that among the Proteobacteria, the most abundant genera were *Klebsiella* (10%), *Helicobacter* (5%), and *Proteus* (2%). In the phylum Firmicutes, the genus *Intestinimonas* (0.7%) stood out, while in the phylum Bacteroidetes, the predominant genera were *Parabacteroides* (12%), *Alloprevotella* (8%), and *Bacteroides* (7%) (Fig 1C). In contrast, the microbiota of the PBS group mice presented a different relative abundance, with the genera *Klebsiella* (1%), *Marinobacter* (1%), *Parasutterella* (1%), *Helicobacter* (1%), *Deltaproteobacteria* (0.8%), and *Candidatus saccharimoni* (0.9%) among the Proteobacteria. In the phylum Firmicutes, the families Ruminococcaceae (2%) and Lachnospiraceae (2%) were found, while in the phylum Bacteroidetes, the most prevalent genera were *Alloprevotella* (5%), *Alistipes* (4%), *Odoribacter* (1%), *Parabacteroides* (1%), and *Bacteroides* (2%) (Fig 1D). During the treatment with AMOX or PBS, the mice were weighed daily. It was observed that the dysbiotic mice lost weight compared to the PBS group, both in the middle of the treatment (day 7) and at the end (days 14 and 15) (S1 Figure).

### 3.2. Amoxicillin-induced bacterial gut dysbiosis shows alterations in the intestinal mucosa and caecum architecture derangement in mice

**Fig 2:**
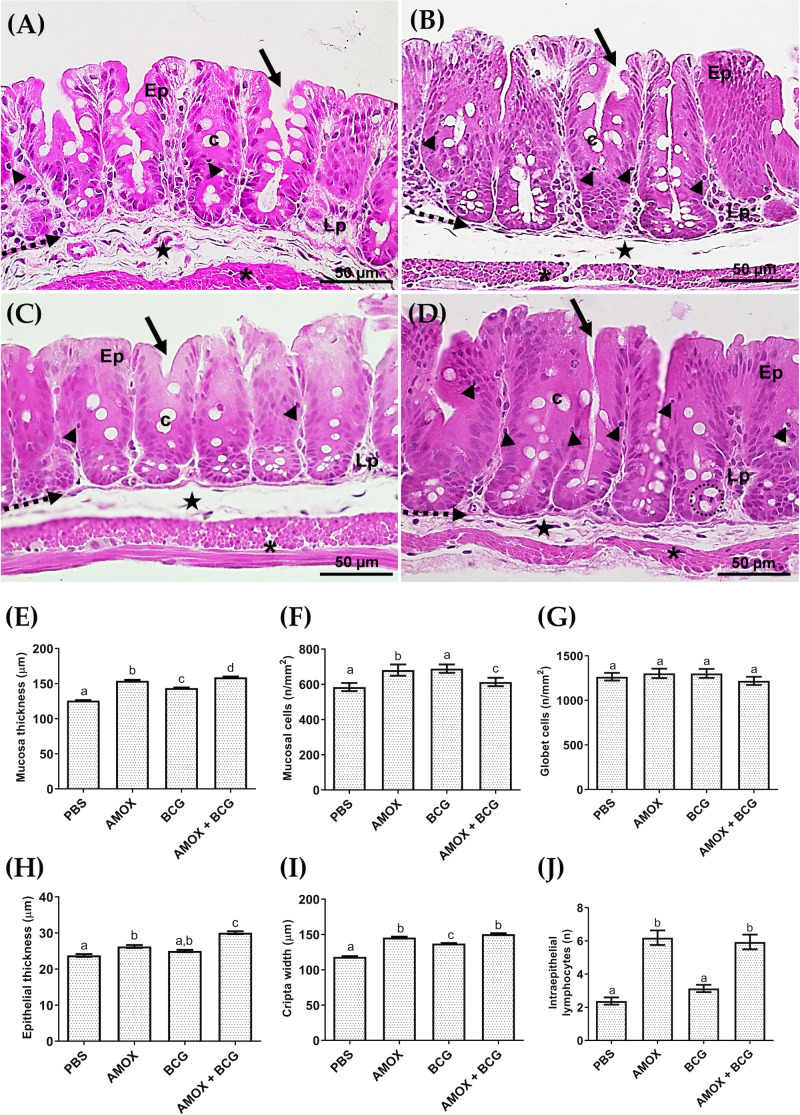
The microstructural analysis of the caecum is altered by amoxicillin-induced bacterial gut dysbiosis in mice. Representative photomicrographs (40×) of the caecum from the (A) PBS, (B) AMOX, (C) BCG, and (D) AMOX +  BCG groups are shown. Quantitative evaluation includes (E) Mucosal thickness (µm), (F) Mucosal cells (n/mm²), (G) Goblet cells (n/mm²), (H) Epithelial thickness (µm), (I) Crypt length (µm), and (J) Intraepithelial lymphocytes (n).

Histopathological evaluation in the amoxicillin-induced dysbiosis focusing on caecum alterations was evaluated (Fig 2). Stereological analyses revealed that the intestinal mucosa thickness was significantly altered in all amoxicillin-treated groups (Fig 2B and 2D) compared to those treated with PBS (Fig 2A). A tendency for increased thickness was noted in the AMOX and AMOX +  BCG groups (Fig 2B and 2D) compared to mice only infected with BCG (Fig 2C and 2E).

Additionally, BCG-infected mice (Fig 2C) showed an increase in the number of mucosal cells compared to the PBS controls (Fig 2A and 2F), while goblet cells remained similar across groups (Fig 2G). Epithelial thickness (Fig 2H) also significantly increased in mice treated with AMOX (Fig 2B) and AMOX +  BCG (Fig 2D) compared to PBS-treated mice (Fig 2A), with the greatest increase observed in the AMOX +  BCG group (Fig 2D).

Mice treated with AMOX (Fig 2B), only infected with BCG (Fig 2C), and AMOX +  BCG (Fig 2D) also exhibited a significant increase in crypt length (Fig 2I) compared to PBS-treated mice (Fig 2A), with a tendency for further increase in the AMOX and AMOX +  BCG groups compared to those only infected with BCG. Finally, a significant increase in intraepithelial lymphocytes in the caecum was observed in the AMOX (Fig 2B) and AMOX +  BCG (Fig 2D) groups compared to the PBS control (Fig 2A) (Fig 2J).

### 3.3. Amoxicillin-induced bacterial gut dysbiosis increases the susceptibility of mice to pulmonary *M. bovis* (BCG) infection and lung damage

**Fig 3:**
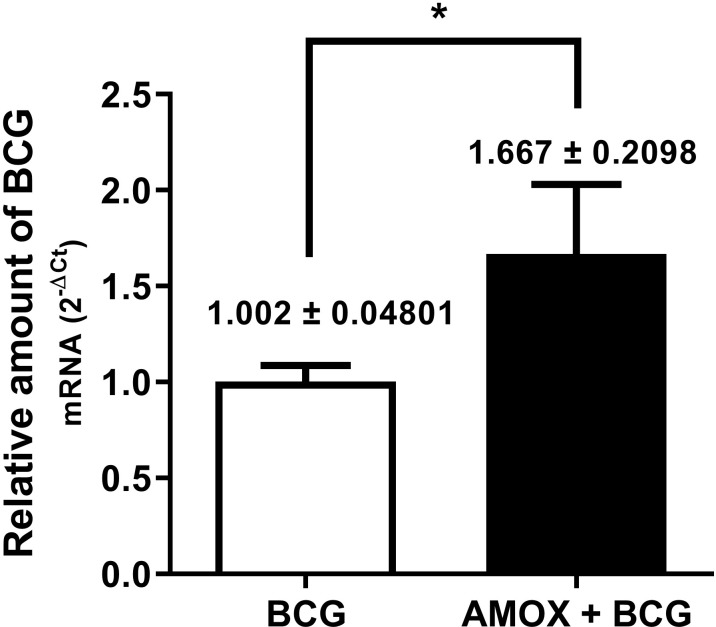
Mice with amoxicillin-induced bacterial gut dysbiosis are more susceptible to *M. bovis*-BCG infection. Relative gene expression of *M. bovi*s-BCG bacteria in the lungs of infected mice (BCG) (white bar) and mice with amoxicillin-induced gut dysbiosis plus *M. bovis*-BCG infection (AMOX +  BCG) (black bar). * p ≤  0.05 related to BCG.

To assess whether amoxicillin-induced gut dysbiosis influences bacterial load in the lungs of BCG-infected mice, relative bacterial gene expression was analyzed by real time RT-PCR. The results (Fig 3) show that mice treated with AMOX and infected with BCG (AMOX +  BCG) exhibited significantly higher bacterial load compared to mice only infected with BCG. These data indicate that amoxicillin-induced gut dysbiosis increases susceptibility to pulmonary infection by *M. bovis*-BCG, resulting in an approximately 67% higher bacterial load compared to mice only infected with *M. bovis*-BCG (Fig 3).

**Fig 4:**
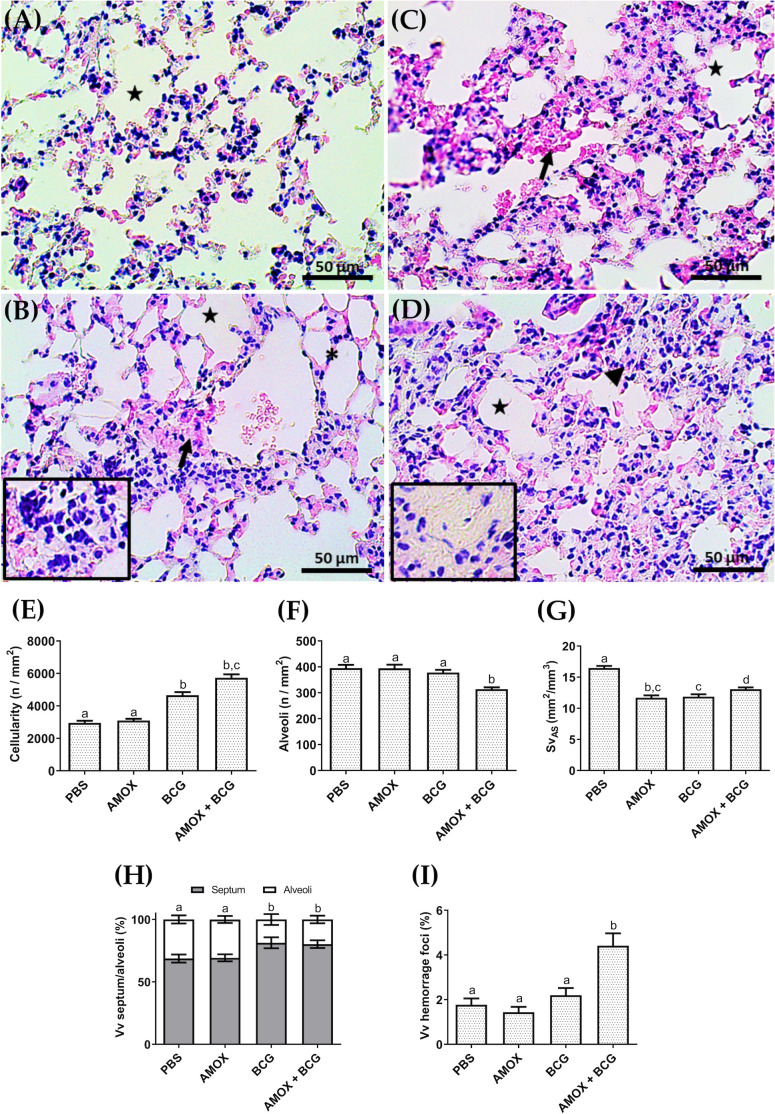
Histopathology and stereological parameters of changes in the lung tissue of C57BL/6 mice. Photomicrographs (40×) show histopathological analyses of the groups: (A) PBS, (B) AMOX, (C) BCG, and (D) AMOX +  BCG. The asterisk indicates normal septum, the arrowhead represents thickened septum, the star marks the alveoli, and the arrow points to alveolar hemorrhage. Additional details show inflammation in AMOX and exudate in AMOX +  BCG. The stereological analysis includes: **(E)** cellularity (n/mm²), **(F)** alveoli (n/mm²), **(G)** alveolar surface density (mm²/mm³), **(H)** septum/alveoli (%), and **(I)** volume of hemorrhagic foci. Treatments with different letters indicate statistically significant differences between them (p ≤  0.05).

After quantifying the bacterial load of *M. bovis*-BCG, histopathological and stereological analyses were performed on the lungs of mice treated or not with amoxicillin and infected or not with *M. bovis*-BCG. In the lungs of PBS-treated mice, the airways and alveoli were unobstructed, with no thickening of the septa and low cellularity (Fig 4A). Mice with amoxicillin-induced gut dysbiosis (AMOX), but not infected, showed some cellularity in isolated regions of the septa (Fig 4B). In contrast, the groups infected with *M. bovis*-BCG (BCG and AMOX +  BCG) exhibited significant cellular influx and reduced alveolar spaces (Fig 4C and 4D). Mice with amoxicillin-induced dysbiosis and infected with BCG (AMOX +  BCG) showed more pronounced inflammatory infiltrates, presence of exudate (Fig 4D highlighted), and significant thickening of the alveolar septa compared to the other groups.

Mice infected with BCG (BCG) and mice with amoxicillin-induced dysbiosis and infected with BCG (AMOX +  BCG) had a higher number of pulmonary inflammatory cells compared to PBS and AMOX controls. However, there was no significant difference between amoxicillin-treated mice (AMOX) and the PBS control (Fig 4E). The alveoli in mice with amoxicillin-induced dysbiosis and infected with BCG (AMOX +  BCG) was reduced compared to all other groups (Fig 4F). The alveolar surface density also decreased significantly in the AMOX, BCG-infected, and AMOX +  BCG-treated groups compared to the PBS controls (Fig 4G). Fig 4H shows that mice infected with BCG and with amoxicillin-induced dysbiosis (AMOX +  BCG) had a reduced alveoli and decreased density of alveolar septa. Dysbiotic mice infected with BCG exhibited a marked increase in the volume of hemorrhagic foci compared to the other groups (Fig 4I).

### 3.4. Amoxicillin-induced bacterial gut dysbiosis decreases *IL-1β* and *IL-6* expression in the lung of *M. bovis* (BCG)-infected mice

**Fig 5:**
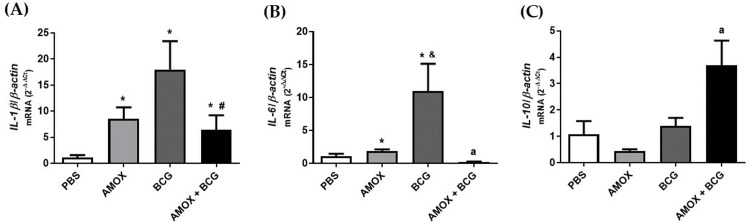
Amoxicillin-induced bacterial gut dysbiosis decreases the expression of (A) *IL-1* *β* and (B) *IL-6* but increases the expression of the (C) *IL-10* gene in the lungs of mice infected with *M. bovis*-BCG. The differential expression of *IL-1β*, *IL-6*, and *IL-10* genes was assessed in the right lung of the PBS, AMOX, BCG, and AMOX +  BCG groups. * p ≤  0.05 related to PBS, ^&^p ≤  0.05 related to AMOX, ^#^p ≤  0.05 related to BCG, and ^a^p ≤  0.05 related to PBS, AMOX, and BCG.

A variation in the relative load of *M. bovis*-BCG and in the histopathological damage of the lungs in mice was observed. To understand these differences, we analyzed the immunological profile of the lungs, focusing on the differential expression of genes encoding pro- and anti-inflammatory cytokines. There was a significant increase in the expression of the pro-inflammatory genes *IL-1β* and *IL-6* in the group with dysbiosis induced by amoxicillin (AMOX) compared to the PBS control group (Figs 5A and 5B). In mice infected only with BCG, an increase in the differential expression of *IL-1β* and *IL-6* was observed. However, in animals with dysbiosis and infection (AMOX +  BCG), there was a decrease in the expression of these genes compared to animals infected only with BCG (Figs 5A and 5B). On the other hand, the expression of the anti-inflammatory cytokine *IL-10* was significantly increased in animals with amoxicillin-induced dysbiosis and infection (AMOX +  BCG) compared to the other groups (Fig 5C). Demonstrating an anti-inflammatory state in the lungs of mice with bacterial gut dysbiosis induced by amoxicillin and infected with *M. bovis*-BCG (AMOX +  BCG).

## 4. Discussion

Several pieces of evidence show that antibiotic use affects bacterial communities, leading to a dysbiotic state associated with metabolic and immunological disorders, as well as increased susceptibility to developing diseases, ultimately compromising long-term health [15]. The present study demonstrated the role of the gut microbiota in response to exposure to *M. bovis-BCG*. It was observed that treatment with amoxicillin led to weight loss in the animals from the fifth day (Fig S1), which was also noted in previous studies with ampicillin administration [16]. Conversely, in studies by Silva and collaborators [10], with female 129Sv/Ev mice strain, amoxicillin treatment induced weight gain, while in the study by Tulstrup and colleagues [17], with Wistar rats, there was no significant weight change. Supporting the present study, Latiu and collaborators [18], observed weight loss in mice treated with amoxicillin for 14 days. Generalized changes in the gut microbiota due to antibiotic use can limit the ability to extract nutrients, leading to calorie loss in the feces and consequent weight loss, highlighting the important role of the microbiome in nutrient metabolism [19]. The effect of antibiotics may vary depending on specific target species, pharmacokinetics, and intestinal absorption, determining whether they have a greater or lesser impact on the host’s overall health [20].

Metagenomic sequencing analysis revealed alterations in bacterial phyla, with an increased relative abundance of Proteobacteria in animals treated with amoxicillin and a decrease in the Bacteroidetes and Firmicutes phyla compared to the control group, which was also observed in the study by Rosa and collaborators [13], in dysbiosis induced by vancomycin. The elevated prevalence of Proteobacteria is a sign of dysbiosis and can contribute to inflammatory processes and increase the risk of developing other pathologies [21]. These commensal Enterobacteriaceae (phylum Proteobacteria), benign in a healthy state, take advantage of and utilize the nitrate generated by the host’s inflammatory process [22] to outgrow Firmicutes and Bacteroidetes, which depend on fermentation for their growth. In studies with pregnant and non-pregnant rats treated with amoxicillin, an increase in Proteobacteria density and a reduction in Firmicutes were also observed [23].

Specific taxa were investigated, and it was possible to observe that among Proteobacteria, the most abundant genera in amoxicillin-treated mice were *Proteus*, *Klebsiella*, *Parabacteroides*, and *Helicobacter*, while control animals had a higher relative abundance of bacteria from the families Ruminococcaceae, Lachnospiraceae, and Rikenellaceae (Fig 1B). C57BL/6 mice exposed to ampicillin for 14 days also had their microbial community altered, with treated animals showing an increased relative abundance of *Klebsiella* [16]. Supporting our studies, Winglee and colleagues [24], and Hanada and collaborators [16], also found higher relative abundances of Ruminococcaceae and Lachnospiraceae. The caecum and colon of rats host the densest and most diverse communities in the body. In the caecum, plant fiber digestion occurs slowly due to the microbiota. The rat caecum is enriched with the Ruminococcaceae and Lachnospiraceae communities, while Rikenellaceae can typically be found in both the caecum and colon [25].

Histological analyses of the caecum demonstrated alterations in the intestinal mucosa of mice treated with AMOX and AMOX +  BCG. A significant increase in caecum epithelial thickness was observed in both groups, more pronounced in the AMOX +  BCG group, as well as a significant increase in crypt length. Amoxicillin, one of the most widely used antibiotics in human and animal health, can alter the intestinal microbial composition, potentially triggering an adaptive epithelial response to maintain intestinal barrier function. Simultaneously, changes in the pulmonary microbiome, including those occurring during bacterial infections, can activate mucosal immunity by stimulating cytokine release and growth factor production. This process may synergistically enhance epithelial cell turnover and influence the gut, leading to crypt elongation, exemplifying the bidirectional nature of the gut-lung axis [26,27]. This result was not observed in a previous study using broad-spectrum antibiotics (ampicillin, neomycin, metronidazole, and vancomycin) for three weeks [28]. The intestinal microbiota participates in protection against the colonization of pathogenic bacteria, impairing their growth and/or producing bacteriocins, in addition to preventing bacterial entry by maintaining the integrity of the intestinal epithelium [29]. In another study, the ileum of animals infected and treated with antibiotics before and after infection with *Mycobacterium tuberculosis* showed distortion of intestinal microvilli, which was restored after fecal transplantation [23].

Goblet cells, a component of the mucous layer, play a role in innate immunity by secreting mucins, providing the first line of defense against physical and chemical damage, and protecting against pathogen invasion [30]. In our studies, there was no difference in the number of goblet cells, but an increase in intraepithelial lymphocytes was observed in animals treated with amoxicillin and AMOX +  BCG, which may be related to immune system stimulation, considering that these represent a heterogeneous population of T cells [31]. Other researchers found significant and extensive caecum mucosa inflammation in mice in response to antibiotic use post-infection with *Salmonella* [32,33]. Another study observed a decrease in distinct immune cells, such as helper T cells, cytotoxic T cells, memory and effector T cells, B lymphocytes, regulatory T cells, and activated dendritic cells [33]. The group that received only BCG without dysbiosis did not show an increase in intraepithelial lymphocytes, suggesting that BCG alone does not induce changes in the gut microbiota.

The lungs from mice in the BCG and AMOX +  BCG groups were analyzed for bacterial load, with the second group showing an increase in load (Fig 3). Supporting this study, an increase in lung bacterial loads was also found six hours after infection in dysbiotic animals with *M. tuberculosis* [23]. This result demonstrates that dysbiosis leads to greater susceptibility to BCG, which is discussed by some authors as the susceptibility to pulmonary infections being dependent on the modulation of the gut microbiota composition in murine models, as it regulates the immune response in the respiratory tract, highlighting the ability of the gut microbiota to modulate immune responses in distant organs [34]. BCG replication is important for its immunomodulatory role [35], but it was observed that in dysbiotic animals, there is an increased expression of bacterial load after treatment with amoxicillin for 15 days, which may be related to immune system manipulation resulting from intestinal dysbiosis.

An increase in pulmonary cellularity was observed, confirmed by stereological analyses of the lungs of animals infected with BCG and AMOX +  BCG. Lung tissue damage characteristic of an inflammatory process was noted, such as alveolar space narrowing and alveolar obstruction, along with a greater presence of inflammatory cell infiltrates compared to control groups. The AMOX +  BCG group, compared to the BCG group, also presented exudate, increased cellularity, and the presence of hemorrhagic foci, more intensely. These results are similar to those previously found when dysbiotic animals infected with *M. tuberculosis* showed a greater number and size of granulomas and a higher bacterial load in other organs, demonstrating the crucial role of the gut microbiota in the response against pulmonary infections [23].

According to the quantification of cytokine gene expression from lung macerates, it was observed that animals infected with BCG showed an increase in the expression of *IL-1β* and *IL-6* genes compared to other groups, while *IL-10* remained the same as the control group. This result demonstrates the pro-inflammatory role of BCG, as its mechanism of action has been attributed to the recruitment of CD4 T cells that produce pro-inflammatory cytokines to combat infection [36]. On the other hand, dysbiotic animals infected with BCG showed lower expression of *IL-1β* and *IL-6* and higher expression of *IL-10* compared to the non-dysbiotic group, meaning they exhibited a reduced pro-inflammatory profile and a greater tendency towards an anti-inflammatory profile mediated by *IL-10*.

According to Pitt and collaborators [37], Roach and collaborators [38], and Redford and collaborators [37], during primary infection with *M. tuberculosis* in vivo, *IL-10* is shown to negatively regulate the immune response. Other studies highlight greater control of *M. tuberculosis* bacterial load in mice with *IL-10* signaling blockade [39,40], which corroborates the results of this study, as the presence of *IL-10* is associated with a high bacterial load and greater tissue damage in the lungs.

Pro-inflammatory interleukins IL-1β and IL-6 recruit immune cells, induce fever, and increase vascular permeability to fight invaders, but they can cause damage if there is an exaggerated response. Thus, anti-inflammatory interleukins like IL-10 help prevent damage and repair tissues by inhibiting pro-inflammatory cytokines and recruiting regulatory cells. However, an exaggerated anti-inflammatory response can also lead to immune suppression and impair pathogen clearance [41].

Therefore, high IL-10 production may be linked to greater damage, as observed in the previously presented histopathological evaluations.The use of antibiotics can disrupt the regulatory pathways of the innate immune response in the lungs and induce intestinal homeostasis disruption, producing a negative impact on *M. tuberculosis*, both at the innate and adaptive immune response levels [42].

However, a significant lung-gut axis relationship was observed, where intestinal dysbiosis caused by amoxicillin altered the immune response profile in relation to pulmonary infection with *M. bovis-BCG*. Further studies are needed to elucidate the specific mechanisms involved in this axis.

## 5. Conclusion

Amoxicillin-induced bacterial gut dysbiosis in C57BL/6 mice led to a significant imbalance in the composition of the intestinal microbiota accompanied by weight loss, histopathological changes in the caecum mucosa and lungs, and an increased susceptibility to pulmonary infection by *M. bovis*-BCG. These findings suggest that amoxicillin-induced dysbiosis may impair the host’s ability to mount an acute effective immune response against pulmonary infections. The impact of antibiotic use, such as amoxicillin, on bacterial gut microbiota and the consequent modulation of the immune response warrants attention, especially in the context of pulmonary bacterial infections. While this study provides important insights into the effects of amoxicillin-induced dysbiosis on immune responses and pulmonary infection, future research could expand on these findings by exploring a broader range of immune and microbial markers, as well as testing in diverse models to further validate and extend our conclusions.

## Supporting information

S1 Figure Mice body weight was evaluated during 14 days of amoxicillin or PBS treatment. * p ≤  0.05 related to PBS.
(TIF)

S1 Table Sequence of primers used to evaluate gene expression. (DOCX)
