# Supplementary figures and images for: Amoxicillin-induced bacterial gut dysbiosis decreases IL-1β and IL-6 expression but exacerbate lung inflammatory response against Mycobacterium bovis—Bacille Calmette-Guérin (BCG)
Source: PLoS One. 2025 Feb 26;20(2):e0319382. doi: 10.1371/journal.pone.0319382 (PMC11864530; doi:10.1371/journal.pone.0319382)

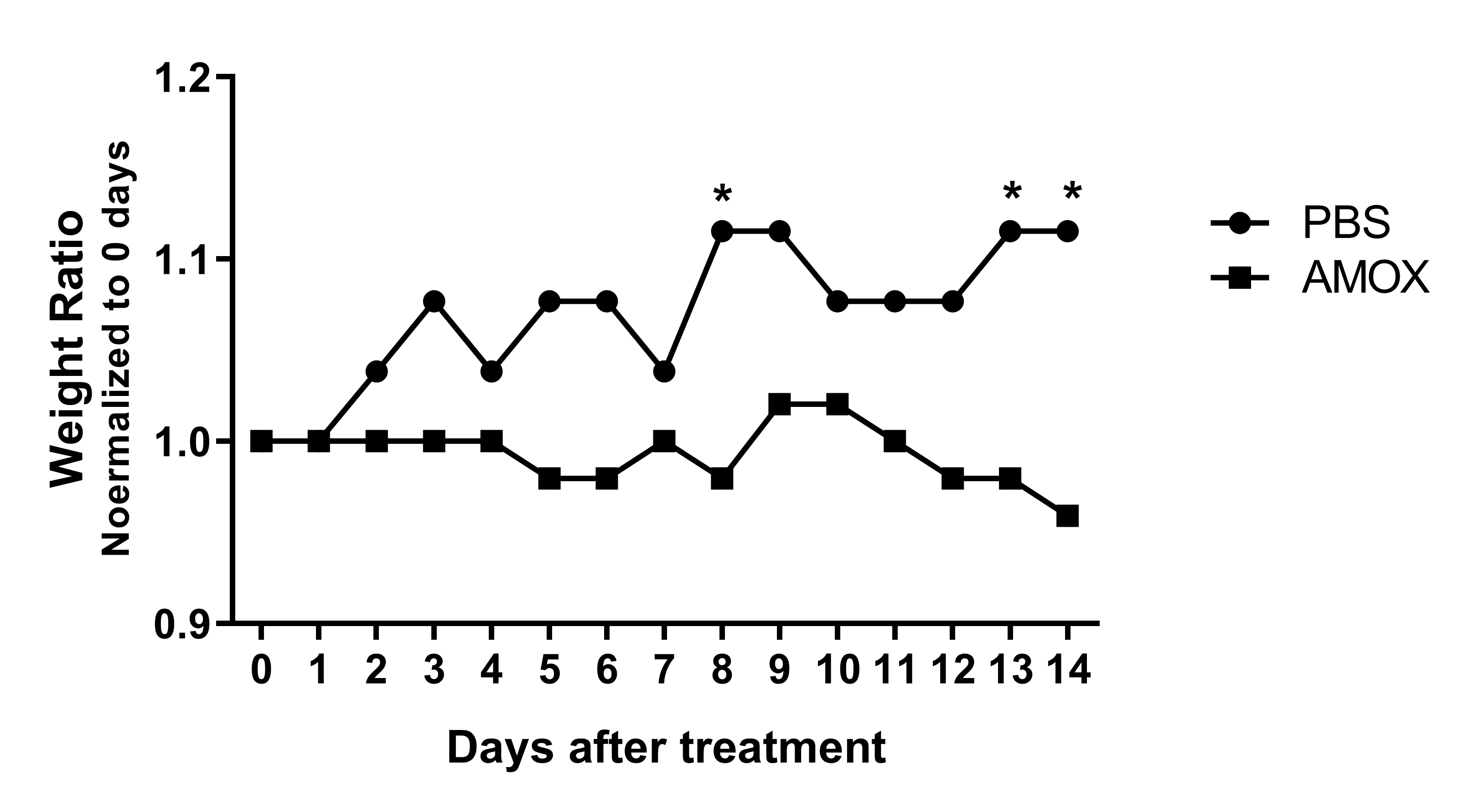

Supplement: S1 Figure — * p ≤ 0.05 related to PBS. (TIF) [file pone.0319382.s001.tif]
