# Supplementary material for: Amoxicillin-induced bacterial gut dysbiosis decreases IL-1β and IL-6 expression but exacerbate lung inflammatory response against Mycobacterium bovis—Bacille Calmette-Guérin (BCG)
Source: PLoS One. 2025 Feb 26;20(2):e0319382. doi: 10.1371/journal.pone.0319382 (PMC11864530; doi:10.1371/journal.pone.0319382)
Supplement: S1 Table — (DOCX) [file pone.0319382.s002.docx]

**Table S1.** Sequence of primers used to evaluate gene expression.

| **Gene** | **Sequence** |
| --- | --- |
| *IL-1β* | F: 5’-TGACCTGGGCTGTCCAGATG-3’  R: 5’-CTGTCCATTGAGGTGGAGAG-3’ |
| *IL-6* | F: 5’-CCAGGTAGCTATGGTACTCCAGAA-3’  R: 5’-GATGGATGCTACCAAACTGGA-3’ |
| *IL-10* | F: 5’-GGTTGCCAAGCCTTATCGGA-3’  R: 5’-ACCTGCTCCACTGCCTTGCT-3’ |
| *BCG* | F: 5’-CCGCCGACCGACCTGACGAC-3’  R: 5’-GGCGATCTGGCGGTTTGGGG-3’ |
| *β-actin* | F: 5’-AGGTGTGCACCTTTTATTGGTCTCAA-3’  R: 5’-TGTATGAAGGTT TGGTCTCCCT-3’ |
